# Nursing and midwifery workforce expansion as a strategic lever to reduce maternal mortality: Global evidence and policy implications from an ecological study

**DOI:** 10.3934/publichealth.2025031

**Published:** 2025-05-28

**Authors:** Wenpeng You

**Affiliations:** 1 Adelaide Medical School, the University of Adelaide, Adelaide, Australia; 2 Adelaide Nursing School, the University of Adelaide, Adelaide, Australia; 3 Heart and Lung, Royal Adelaide Hospital, Adelaide, Australia

**Keywords:** nursing-midwifery workforce, maternal mortality ratio, global health disparities, healthcare workforce shortage, health workforce policy

## Abstract

**Background:**

This global cross-sectional study analyzed data from 266 “countries” and territories to evaluate the relationship between the nursing and midwifery workforce size (NMWS) and maternal mortality ratios (MMR). Drawing from five major United Nations and World Bank databases, the study offers robust and generalizable insights across diverse health systems and economic settings.

**Methods:**

The study examined the association between the NMWS and MMR using scatterplots, bivariate and partial Pearson correlation coefficients, and multiple and stepwise linear regression models. Key confounding variables, including economic affluence measured by the gross domestic product (GDP) per capita adjusted for purchasing power parity, total fertility rate, and urbanization, were included to isolate the independent contribution of the NMWS to maternal health outcomes at the global and regional levels.

**Results:**

The NMWS accounted for 49.13 percent of the global variation in maternal mortality ratios, which indicates a strong inverse relationship. After adjusting for economic and demographic variables, the NMWS remained a significant independent predictor and explained 11.09 percent of the variance. A stepwise regression identified the NMWS as the second most influential predictor of maternal mortality after economic affluence and the fertility rate. The association was strongest in low- and middle-income countries, where workforce shortages and the maternal mortality rates are highest.

**Conclusions:**

This study identifies the NMWS as a critical and measurable factor in reducing maternal mortality worldwide. This study's findings provide compelling evidence for a strategic investment in the nursing and midwifery workforce. Expanding this workforce is essential to improve the maternal health outcomes, especially in countries with limited resources, and should be prioritized in global maternal health and workforce planning strategies.

## 1. Background

Reducing maternal Mortality remains a global health priority and a critical indicator of the healthcare system's performance and equity. The maternal mortality ratio (MMR), defined as the number of maternal deaths per 100,000 live births, encompasses fatalities during pregnancy or within 42 days of the termination of a pregnancy due to complications related to or aggravated by pregnancy or its management [1]. Although the global maternal deaths decreased from 532,000 in 1990 to 303,000 in 2015 [2], the recent stagnation and rising maternal deaths in some regions underscore the urgent need for renewed efforts and strategic investments [3].

Low- and middle-income countries (LMICs) continue to bear the brunt of maternal deaths, thereby accounting for approximately 94% of the global maternal mortality [2],[4]. These deaths often result from preventable causes such as postpartum hemorrhage, infections, hypertensive disorders, and unsafe abortion [3],[5]. Timely access to skilled birth attendants, especially nurses and midwives, can prevent most of these outcomes [6],[7]. Despite ongoing improvements in maternal health services, disparities in outcomes between LMICs and high-income countries (HICs) persist, with structural healthcare system limitations and workforce shortages playing a major role.

The nursing and midwifery workforce is central to maternal care delivery across the continuum, encompassing antenatal, intrapartum, and postnatal care. The availability, accessibility, and quality of this workforce directly influence maternal survival. Skilled nursing and midwifery professionals are trained to identify complications, manage obstetric emergencies, and provide life-saving interventions for mothers and newborns [6],[7]. Their role becomes especially crucial in under-resourced settings where physicians are scarce and access to advanced care is limited.

Growing evidence supports the relationship between the nursing-midwifery workforce size (NMWS) and maternal health outcomes, particularly in LMICs [8]–[10]. Increases in the NMWS have been associated with improved maternal health indicators, reduced neonatal mortality, and better health service coverage [11],[12]. However, most previous studies have relied on individual-level data or qualitative assessments, and few have offered a robust global analysis that adjusts for confounding factors such as economic affluence, the total fertility rate (TFR), and urbanization. These factors significantly shape maternal health outcomes by influencing the service delivery capacity and the population health risks [11]–[14].

Workforce shortages, particularly among nurses and midwives, represent a persistent and growing barrier to achieving maternal health goals. The World Health Organization (WHO) estimates a global shortfall of 900,000 midwives, with the highest deficits in sub-Saharan Africa and South Asia, which are regions where the MMRs remain alarmingly high [15],[16]. In addition to quantity, quality is essential. Workforce readiness depends on adequate education, professional regulation, and opportunities for continuing development. Midwives must be trained in accordance with international standards, such as those established by the International Confederation of Midwives (ICM), which emphasize core competencies, ethical practices, and autonomy [16].

The COVID-19 pandemic exacerbated the existing challenges, thereby placing unprecedented stress on healthcare systems and widening inequalities in maternal care. Nurses and midwives experienced heightened emotional exhaustion, increased workloads, and workplace violence, thus contributing to burnout and attrition [17],[18]. Although this study does not focus on pandemic-specific dynamics, its long-term consequences have intensified workforce challenges, thus reaffirming the urgency of strategic workforce planning.

Health system factors such as governance structures, professional autonomy, and interprofessional collaborations significantly influence how midwives and nurses contribute to maternal care [19]. In many settings, midwifery remains subordinate to physician-led models, which restricts midwives from practicing to their full capacity [20],[21]. In contrast, shared leadership models that promote midwifery autonomy have been associated with better maternal outcomes and more efficient care delivery [20],[21]. As countries aim to reduce maternal mortality, it is essential to consider both the size of the workforce and the extent to which nurses and midwives are integrated into the health system. Research by Downe and colleagues highlighted that the maternal outcomes improved and the overall system effectiveness increased when midwives were supported to practice autonomously within their collaborative teams [22].

This study addresses a significant knowledge gap by conducting a comprehensive global cross-sectional analysis of the relationship between the NMWS and the MMR. Using data from 266 countries and territories sourced from five major United Nations and World Bank databases, it extends beyond prior research that has largely focused on descriptive trends or single-country analyses. The study employs advanced statistical techniques, beginning with a scatter plot analysis to visualize the confounded relationship between the NMWS and the MMR. Then, it applies a partial correlation analysis, as well as multiple and stepwise linear regression models, to quantify the independent contribution of the NMWS to maternal mortality outcomes after adjusting for key confounders such as economic affluence, the TFR, and urbanization [11]–[13].

By integrating these variables into the analysis, this study offers a more nuanced understanding of the relative contribution of the NMWS to maternal mortality. Additionally, it examines how the association varies across regions and income groups, with a particular emphasis on LMICs, where workforce shortages are the most acute and where scaling the nursing and midwifery workforce could have the greatest impact. The study's findings aim to inform global and national workforce policies and maternal health strategies by providing robust evidence on the role of nursing and midwifery workforce expansion in reducing maternal deaths.

## 2. Methods and materials

### 2.1. Data sources

This study utilized data from five major population-level datasets compiled by United Nations agencies to investigate the relationship between the NMWS and maternal mortality. By analyzing 266 reporting units, including countries and territories from across the globe, this study provides one of the most comprehensive assessments to date on the association between the workforce density and maternal health outcomes. Its wide geographic and economic coverage enhances the generalizability of findings and enables meaningful cross-regional comparisons. These insights are particularly valuable for global health workforce planning and maternal health policies, especially in the context of persistent disparities in maternal mortality across income levels and regions.

The key independent variable, the NMWS, is defined as the number of nurses and midwives per 1000 population. To ensure reliability, this measure was averaged over a five-year period from 2014 to 2018, which helps to smooth short-term fluctuations. The NMWS data were obtained from the World Bank Data Bank and related UN databases [23],[24], and align with established global studies that use the workforce density as an indicator of health system capacity [25],[26]. This metric reflects the availability of skilled health professionals capable of delivering essential maternal care services, making it a robust measure to evaluate maternal health outcomes across diverse settings.

The dependent variable, MMR, is defined as the number of maternal deaths per 100,000 live births due to pregnancy-related causes that occur during pregnancy or within 42 days of termination [2]. The MMR data were obtained from the World Bank and other UN agencies, which are widely recognized for their utility in monitoring global progress toward maternal health goals, guiding international comparisons, and informing national policy planning.

These maternal mortality data provide the foundation for evidence-based resource allocation and strategy development at the global level. Understanding how factors such as the workforce density, economic development, fertility rates, and urbanization contribute to the MMR is critical to shape effective interventions and policy responses.

There is increasing recognition that the current WHO definition of maternal mortality, which captures deaths up to 42 days postpartum, may underestimate the true burden. Efforts are underway to expand the definition to include deaths up to one year postpartum to reflect delayed complications and broader maternal health risks. This potential shift may influence future estimates, surveillance frameworks, and intervention strategies aimed at improving maternal health.

To better capture these dynamics, several confounding variables were included. Economic affluence was measured using the gross domestic product per capita adjusted for purchasing power parity (GDP PPP) in international dollars and using 2014 data. Economic prosperity is consistently associated with improved maternal outcomes through expanded access to healthcare, sanitation, immunization, and adequate nutrition for mothers and infants [27]–[29].

Urbanization is measured as the percentage of the population living in urban areas in 2014. Urban settings typically offer improved infrastructure, access to healthcare facilities, housing, and education, all of which are positively correlated with reduced maternal mortality [30],[31].

The TFR, which is defined as the average number of children a woman is expected to have over her lifetime, was also included. High fertility rates can increase maternal health risks through repeated pregnancies, heightened nutritional demands, and a greater exposure to perinatal complications. Additionally, the TFR serves as an indirect measure of healthcare access and the availability of reproductive services [32]–[34].

The GDP PPP, TFR, and urbanization data were all collected for 2014 to align with the five-year average period used for the NMWS, thus ensuring consistency across the dataset and comparability across the models employed.

### 2.2. Data availability

The data used in this study were obtained from publicly accessible repositories managed by international organizations, as outlined in the “Materials and Methods” section along with the relevant references. These datasets were accessed in compliance with the public usage policies of the respective agencies, and no formal authorization was required for academic use. As the data are fully anonymized and contain no identifiable information pertaining to individuals, families, or communities, this research did not require ethical approval or informed consent.

### 2.3. Data preparation

A comprehensive dataset comprised of 266 reporting units, which is referred to in this study as countries or populations, was compiled and classified according to the World Bank income categories. All study variables were sourced from the World Bank data repository and organized using Microsoft Excel® to ensure consistency across analyses. Due to variations in data availability and reporting across countries, some variables were missing for certain units. As a result, the sample size varied across different analytical models, thereby reflecting these inconsistencies.

### 2.4. Multicollinearity check

Multicollinearity is a frequent issue that can affect the quality of data in the regression analysis. It arises when independent and confounding variables are strongly correlated, which may undermine the reliability of the regression outcomes. To address this, multicollinearity was examined by calculating the correlations between five variables (NMWS, MMR, economic affluence, TFR, and urbanization) using a standard multiple linear regression model with the enter (full entry) method. Multicollinearity was deemed not to be a concern, as the tolerance values were ≥0.10 and the variance inflation factor (VIF) values were ≤10 in both diagnostic tests [35].

### 2.5. Statistical analysis approaches

To comprehensively assess the relationship between the NMWS and the MMR, analyses were conducted at three levels: accounting for confounding factors, isolating independent effects, and determining whether the NMWS serves as a key influencing variable. This evaluation followed a systematic five-step framework that utilized six analytical methods [36]:

1. Scatter plots were generated using Microsoft Excel® 2016 to visually depict the strength, direction, and trend of the relationship between the NMWS and the MMR.
2. Bivariate correlation analyses were globally conducted using Pearson's r for linear relationships and non-parametric methods for monotonic relationships. This method facilitated the identification of associations between all five variables, irrespective of their distribution type.
3. A partial correlation analysis was conducted to identify the independent relationships between the variables. Each variable (NMWS, economic affluence, urbanization, and TFR) was examined as a standalone predictor, while the remaining three were considered as potential confounders. Additionally, this approach assessed the influence of adjusting for individual variables on the relationship between the NMWS and the MMR.
4. A multiple linear regression (enter method) was applied to assess the combined influence of the NMWS and confounders (economic affluence, TFR, and urbanization) on the MMR. Separate analyses were conducted to compare models that included or excluded the NMWS as a predictor. Additionally, a stepwise regression was employed to rank the variables based on their predictive significance for MMR in both models.
5. A correlation analysis across country classifications was conducted to explore the consistency of NMWS-MMR relationships across various groupings. Countries were categorized using criteria such as the following:
  - World Bank income classifications (low-income, lower-middle-income, upper-middle-income, and high-income), and in alignment with the WHO's focus on maternal mortality disparities in LMICs [37], Fisher's *r*-to-*z* transformations were applied to assess differences in correlations between LMICs and high-income nations.
  - Development status (developed vs. developing) based on United Nations criteria [38], with similar comparisons of NMWS-MMR correlations using Fisher's *r*-to-*z* transformations.
  - WHO regional classifications (e.g., Africa, Americas, Europe) [39].
  - Economic and cultural groupings, including regional alliances and economic blocs such as the Asia-Pacific Economic Cooperation (APEC), Asia-Pacific Economic Cooperation (OECD), and Arab World nations [40]–[47].

This comprehensive approach sought to offer a detailed analysis of the impact of the NMWS on the MMR, thereby considering variations across economic, cultural, and geographical contexts.

For the analysis, bivariate correlations, partial correlations, and multiple linear regression models (both enter and stepwise) were carried out using SPSS v. 29. Statistical significance was set at 0.05, with additional thresholds at 0.01 and 0.001. The regression models adhered to strict inclusion criteria, thereby requiring an entry probability (*F*) ≤ 0.05 and a removal probability (*F*) ≥ 0.10. These methodologies provided a thorough and robust assessment of the relationships between the variables and their collective impact on MMRs.

## 3. Results

The scatterplot demonstrates a robust, statistically significant, negative logarithmic relationship between the NMWS and the MMR, with a correlation coefficient of *r* = −0.701 (*R²* = 49.13%, *p* < 0.001, *n* = 231). This suggests that the NMWS accounts for around 49.13% of the variation in the MMR on a global scale. A higher concentration of nursing and midwifery professionals per 1000 people is associated with a significant reduction in maternal mortality, thus highlighting the vital role that the workforce size plays in shaping maternal health outcomes across regions. The substantial impact of the NMWS on the MMR stresses the importance of targeted investments in the nursing and midwifery workforce, particularly in areas where the workforce density is currently insufficient.

**Figure 1.**
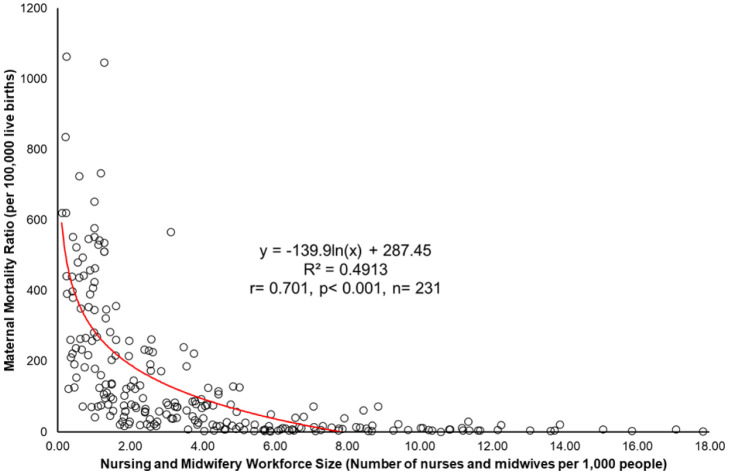
A plot which illustrates that the size of the nursing and midwifery workforce size accounts for nearly 50% of the global variation in maternal mortality ratios.

Data source and definitions: The NMWS is calculated as the number of nurses and midwives per 1000 population, sourced from the World Bank. The MMR represents the number of maternal deaths per 100,000 live births due to pregnancy-related causes, which occur during pregnancy or within 42 days post-pregnancy termination, as reported by the World Bank.

Table 1 highlights a strong inverse relationship between the NMWS and the MMR on a global scale, with Pearson's *r* = −0.812 and Spearman's *ρ* = −0.834, both showing a high statistical significance (*p* < 0.001). Moreover, economic affluence, which is represented by economic affluence, and urbanization were found to negatively correlate with the MMR in both parametric and nonparametric analyses, thus suggesting that improved economic conditions and urban living environments contribute to a reduced maternal mortality. Conversely, the TFR showed a significant positive association with the MMR, thus indicating that higher fertility rates are linked to an elevated maternal mortality, likely due to increased health risks and resource constraints associated with higher numbers of pregnancies.

**Table 1.** Variable Correlation Matrix (Pearson's Correlations Displayed Above the Diagonal, Non-Parametric Correlations Below).

Project	Nursing and midwifery workforce size	Maternal mortality ratio	GDP PPP	Total fertility rate	Urbanization
Nursing and midwifery workforce size	1.000	−0.812**	0.830**	−0.761**	0.568**
Maternal mortality ratio	−0.834**	1.000	−0.840**	0.786**	−0.562**
GDP PPP	0.836**	−0.853**	1.000	−0.828**	0.693**
Total fertility rate	−0.747**	0.800**	−0.811**	1.000	−0.497**
Urbanization	0.610**	−0.614**	0.732**	−0.504**	1.000

Note: Significance level: **p < 0.001; Sample size range: 225–263. Data Sources and Definitions: The per capita GDP, adjusted for the PPP and presented in international dollars, reflects the economic output per person, based on the total value of goods and services produced annually. Urbanization is defined as the percentage of the population that resides in urban areas, sourced from the World Bank. All datasets were log-transformed for consistency in the correlation analyses.

Table 2 reveals that the NMWS maintained a significant inverse correlation with the MMR (*r* = −0.333, *p* < 0.001) when controlling for economic affluence, the TFR, and urbanization, thus underscoring its critical role in reducing maternal mortality. This finding indicates that NMWS independently explained approximately 11.09% of the variation in the MMR, even after adjusting for these confounding variables.

Additional analyses, where economic affluence, the TFR, and urbanization were individually controlled, confirmed the robustness of this relationship. In all scenarios, the NMWS consistently showed an inverse association with the MMR (*r* = −0.380, −0.533, and −0.724, *p* < 0.001 for each). Additionally, economic affluence demonstrated an independent and significant inverse correlation with the MMR (*r* = −0.508, *p* < 0.001), further highlighting its importance as a determinant of maternal health outcomes. These results highlight the complementary yet distinct contributions of the NMWS and economic factors in addressing maternal mortality (Table 2).

**Table 2.** Partial correlation analysis of maternal mortality ratio with nursing and midwifery workforce size, GDP PPP, total fertility rate, and urbanization.

Variables	Partial correlation analysis of maternal mortality ratio with nursing and midwifery workforce size, GDP PPP, total fertility rate, and urbanization as predictors, with other variables controlled.											
	Maternal mortality ratio			Maternal mortality ratio			Maternal mortality ratio			Maternal mortality ratio		

	*r*	*p*	*df*	*r*	*p*	*df*	*r*	*p*	*df*	*r*	*p*	*df*
Nursing and midwifery workforce size	−0.333	<0.001	218	-	-	-	-	-	-	-	-	-
GDP PPP	-	-	-	−0.286	<0.001	218	-	-	-	-	-	-
Total fertility rate	-	-	-	-	-	-	0.272	<0.001	218	-	-	-
Urbanization	-	-	-	-	-	-	-	-	-	−0.004	0.949	218
Nursing and midwifery workforce size	-	-	-	−0.380	<0.001	222	−0.533	<0.001	228	−0.724	<0.001	228
GDP PPP	−0.508	<0.001	222	-	-	-	−0.545	<0.001	222	−0.755	<0.001	222
Total fertility rate	0.444	<0.001	228	0.300	<0.001	222	-	-	-	0.706	<0.001	230
Urbanization	−0.208	<0.010	228	0.051	0.445	222	−0.318	<0.001	230	-	-	-

Tables 3–1 and 3–2 illustrate the relationships between the NMWS and the MMR using standard multiple linear regression models. In the initial regression analysis (Table 3–1), where the NMWS was excluded, economic affluence and the TFR emerged as significant predictors, with beta coefficients of *β* = −0.594 and *β* = 0.308, respectively (*p* < 0.001). When the NMWS was included in the model, it showed a significant inverse association with the MMR (*β* = −0.312, *p* < 0.001), while economic affluence and the TFR remained significant predictors, with adjusted beta values of −0.364 and 0.256, respectively (*p* < 0.001).

In the stepwise regression analysis (Table 3–2), economic affluence and the TFR were identified as key predictors when the NMWS was not included, with adjusted *R²* values of 0.704 and 0.732, respectively. When the NMWS was incorporated into the model, it emerged as the second most significant predictor, thereby boosting the adjusted *R²* value to 0.744. The inclusion of the TFR as the final variable further enhanced the model's explanatory power, which resulted in an adjusted *R²* of 0.762. Collectively, these findings demonstrate that the NMWS, along with economic affluence and the TFR, plays a significant role in explaining 76.2% of the variation in the MMR, thus highlighting its critical influence on maternal health outcomes.

**Table 3.** Multiple linear regression analysis which highlights the predictive influence of independent variables on the MMR, with and without the inclusion of the NMWS as a predicting factor.

3–1 Enter regression analysis of maternal mortality ratio with and without the inclusion of nursing and midwifery workforce size as a predictor.				
	Maternal mortality ratio			
	Nursing and midwifery workforce size (not added)		Nursing and midwifery workforce size (added)	

Variable	*Beta*	*Sig*.	*Beta*	*Sig*.
Nursing and midwifery workforce size	-	-	−0.312	<0.001
GDP PPP	−0.594	<0.001	−0.364	<0.001
Total fertility rate	0.308	<0.001	0.256	<0.001
Urbanization	0.016	0.743	−0.003	0.949

3–2 Stepwise Regression Analysis of Maternal Mortality Ratio with Variables Entered Sequentially, Including and Excluding Nursing and Midwifery Workforce Size				
	Maternal mortality ratio			
	Nursing and midwifery workforce size (not added)		Nursing and midwifery workforce size (added)	

Model	Variable	Adjusted *R^2^*	Variable	Adjusted *R^2^*

1	GDP PPP	0.704	GDP PPP	0.704
2	Total fertility rate	0.732	Nursing and midwifery workforce size	0.744
3	Urbanization	Insignificant	Total fertility rate	0.762
4	Nursing and midwifery workforce size	Not added	Urbanization	Insignificant

Table 4 summarizes the relationships between the NMWS and the MMR across 25 country classifications, with 50 correlations analyzed, all of which were negative. Notably, 47 of these correlations were statistically significant, even in instances where the sample sizes were relatively small, thus reaffirming the protective role of the NMWS in lowering the MMR across different economic and regional contexts.

**Table 4.** Bivariate analysis of the correlation coefficient between nursing and midwifery workforce density and maternal mortality ratios across diverse country groupings.

Country groupings	Nursing and midwifery workforce size to maternal mortality ratio			Means of independent and dependent variables	

	Pearson's *r*	Non-parametric	*n*	Nursing and midwifery workforce	Maternal mortality ratio
Worldwide	−0.812***	−0.834***	231	4.190	153.627
World bank income classifications			
Low income	−0.610***	−0.584**	26	0.813	455.654
Lower middle income	−0.507***	−0.498***	51	1.377	202.255
Upper middle income	−0.519***	−0.527**	50	4.024	64.88
High income	−0.611***	−0.500***	56	8.056	14.947
Low- and lower-middle income	−0.489***	−0.735***	127	2.538	200.047
Fisher's *r*-to-*z* transformation: Low- and lower-middle income *vs*. High income	*z* = 1.07, *p* = 0.142	*z* = −2.38, *p* < 0.010			
UN common practice			
Developed	−0.273	−0.439**	45	9.055	7.957
Developing	−0.737***	−0.752***	140	2.844	186.936
Fisher's *r*-to-*z* transformation:Means of independent and dependent variablesDeveloping *vs*. Developed	*z* = −3.76, *p* < 0.001	*z* = −2.87, *p* < 0.010			
WHO Regions			
AFRO	−0.555***	−0.481***	47	1.366	382.064
AMRO	−0.684***	−0.672***	33	3.904	79.118
EMRO	−0.860***	−0.806***	21	2.729	120.619
EURO	−0.381**	−0.301*	49	8.244	11.653
SEARO	−0.362	−0.383	11	2.597	95.545
WPRO	−0.852***	−0.827***	23	4.662	74.565
Countries grouped based on various factors			
ACD	−0.787***	−0.751***	34	3.941	79.500
APEC	−0.769***	−0.754***	19	6.195	43.368
Arab world	−0.792***	−0.811***	20	2.769	123.450
EEA	−0.475**	−0.354	29	8.901	8.379
EOL	−0.818***	−0.855***	50	4.497	185.380
EU	−0.381**	−0.301*	49	8.313	8.815
LA	−0.664***	−0.677***	20	2.952	88.870
LAC	−0.618***	−0.618***	32	3.430	81.364
OECD	−0.604***	−0.338*	37	9.172	10.676
SADC	−0.492*	−0.467	16	2.176	252.313
SCO	−0.750***	−0.597***	26	4.294	73.115

Note: Significance level: * *p* < 0.05; ** *p* < 0.01; ***p < 0.001.

The analysis further indicates that the association between the NMWS and the MMR was more pronounced in developing nations compared to developed ones, which aligns with the WHO's findings that maternal mortality is disproportionately higher in LMICs. Specifically, the NMWS exhibited significantly stronger correlations with the MMR in developing countries (*z* = −3.76, *p* < 0.001 for Pearson's *r*; *z* = −2.87, *p* < 0.01 for non-parametric analysis). A similar pattern was observed when comparing LMICs to high-income nations, with stronger NMWS-MMR associations evident in LMICs (*z* = 1.07, *p* = 0.142 for Pearson's *r*; *z* = −2.38, *p* < 0.01 for non-parametric analysis). These results underscore the critical importance of expanding the NMWS in resource-limited settings, where the impact of workforce shortages on maternal health outcomes is most severe.

## 4. Discussion

Multiple factors have contributed to the global decline in the MMR, among which the expansion of the NMWS appears to be a key determinant. A substantial body of evidence supports the association between the increased availability of skilled nurses and midwives and improved maternal and neonatal outcomes, particularly in low-resource settings [15],[48]. Building upon this foundation, the present study provides a comprehensive global analysis of the relationship between the NMWS and the MMR. Drawing upon data from 266 countries and territories, the study employs a suite of statistical techniques to assess the consistency and strength of this association, while accounting for major economic and demographic confounders.

The results demonstrate a robust and statistically significant inverse association between the NMWS and the MMR. Countries with higher densities of nursing and midwifery professionals reported substantially lower maternal mortality rates. Notably, the NMWS alone accounted for 49.13 percent of the global variation in the MMR. After adjusting for economic affluence, the TFR, and urbanization, the NMWS remained an independent and significant predictor of maternal mortality, which explains 11.09 percent of the variance. The stepwise regression analyses further identified the NMWS as the second most influential predictor, following economic affluence and preceding the TFR, with urbanization contributing comparatively less. The association was particularly strong in LMICs, where persistent workforce shortages continue to hinder progress in maternal health.

This study's extensive geographical coverage and reliance on harmonized data from five major international databases strengthened the validity and generalizability of its findings. To date, it represents one of the most inclusive global investigations into the contribution of the NMWS to maternal mortality reduction and provides critical insights for guiding future workforce development and maternal health policies at the national and international levels.

Expanding the nursing and midwifery workforce is not only a matter of health system capacity but also a fundamental intervention to improve the maternal outcomes. Although centered on participatory women's groups, the intervention was facilitated by community health workers with nursing and midwifery training, who provided structured education on pregnancy care, safe delivery, and neonatal health [49]. This highlights the critical role of midwives and nurses in delivering community-based maternal care and preventive interventions in underserved settings [50],[51]. The WHO recommends scaling up such community-based models in similar settings [52]. Downe et al. and others have shown that midwifery-led continuity models and enabling environments reduce maternal and neonatal mortality, especially in low-resource settings [53]. These models promote respectful, person-centered care and are linked to fewer interventions and better outcomes [54]. Additionally, evidence from LMICs highlights the effectiveness of health worker interventions in improving maternal and infant health and reducing disparities [55]. Nigeria's Abiye Project significantly lowered maternal mortality through community-based care and gained global recognition [56]. Similarly, community-driven programs in high-income countries have helped reduce maternal health inequities [57].

In institutional settings, nurses and midwives are essential to manage childbirth and complications. Research links higher staffing levels to lower maternal mortality, particularly for high-risk pregnancies. In the United Kingdom, specialized nursing care has substantially reduced maternal complications, although recent cuts have threatened these gains. Reviews published in BMC Public Health and Pediatric Nursing confirmed that midwives improve maternal outcomes throughout pregnancy and childbirth, with higher staffing levels associated with fewer maternal and neonatal deaths [58],[59].

The WHO estimates that midwifery-led care, including family planning and skilled birth attendance, could save up to 4.3 million lives annually by 2035 [57],[58],[60],[61]. This projection aligns with findings by ten Hoope-Bender et al. [49], who demonstrated that scaling up midwifery services, particularly in resource-limited settings, substantially improves maternal and newborn health outcomes. Moreover, their work highlights the importance of strong health systems and professional recognition of midwives as central to effective maternal care. At the national level, Nigeria's Abiye (Safe Motherhood) Project exemplifies how expanded access to midwifery care can lead to significant reductions in maternal mortality [56].

However, many prior studies failed to control for socio-economic confounders, thus risking an overestimation of the protective effect of NMWS. As the 2030 Sustainable Development Goals deadline approaches, many countries, particularly in Africa, struggle to meet the target of fewer than 70 maternal deaths per 100,000 live births [62]. The maternity period, which includes pregnancy, childbirth, and postpartum, is a time of heightened vulnerability. Nurses and midwives provide essential services during this period, especially in rural and underserved communities. Training and equitably deploying these professionals can substantially support progress toward the SDGs.

This study builds on prior work by including data from diverse healthcare systems across 266 countries and territories, thereby offering a more global perspective. It adds to the field by examining not only the direct relationship between the NMWS and the MMR, but also the independent effect of the NMWS after adjusting for confounding variables. The analysis found that the NMWS remained a significant predictor of the MMR, even after controlling for economic affluence, the TFR, and urbanization. This underlines the importance of staffing in global maternal health strategies.

Additionally, the study highlights the importance of adequate staffing to prevent iatrogenic complications. Overburdened nurses and midwives are more likely to deliver suboptimal care, including practices that may be perceived as disrespectful or harmful. Such experiences can undermine maternal health and dignity. Ensuring adequate staffing and supportive working environments are essential to uphold the principles of respectful maternity care [63]. Recent evidence has synthesized a century of evolving discourse on respectful care, thus linking workforce conditions and care quality to women's dignity and autonomy in childbirth [64],[65].

The global health workforce shortage is most acute in nursing and midwifery, thus accounting for over half of the deficit [66]. Scatterplot analyses confirmed a strong negative association between the NMWS and the MMR across country classifications, thus underscoring the severity of workforce shortages in LMICs. The WHO reports reinforce that nursing gaps are most pronounced in developing nations, where the maternal mortality is highest.

Despite these challenges, nurses and midwives continue to provide person-centered care across primary, secondary, and tertiary settings. Our findings highlight their protective effect on the MMR, even after accounting for confounding factors. However, the workforce quantity must be matched by the quality [48]. Nurses and midwives must be trained to internationally accepted standards, such as those set by the International Confederation of Midwives, which emphasize ethics, core competencies, and professional regulation [67].

Workforce expansion efforts in LMICs must also confront the challenge of international migration, or “brain drain”. Many trained nurses and midwives leave their home countries in search of better compensation, working conditions, and professional opportunities, which exacerbates the existing workforce shortages [68]. This global mobility undermines national efforts to strengthen maternal healthcare systems. For example, in Nigeria, the Nursing and Midwifery Council has proposed regulatory measures to curb outward migration, thus sparking important ethical debates around balancing national workforce retention with individual autonomy and the right to migrate.

In the subgroup analyses, 44 out of 52 bivariate correlations between the NMWS and the MMR were moderate to strong. These findings affirm the WHO's view of global nursing shortages as a critical challenge that spans both high- and low-income countries. Reducing maternal mortality requires an equitable distribution of nurses across public and private health systems. Programs such as Ghana's National Health Insurance Scheme illustrate the importance of expanding access to skilled birth attendants, although persistent workforce gaps remain [69].

Policy implications are especially relevant for LMICs, where the maternal mortality remains high and health professionals face severe strain [70]. Understanding the extent to which the NMWS influences the MMR can inform targeted investments in training, recruitment, and retention. Additionally, supporting the physical and emotional well-being of nursing staff is essential to sustain their ability to deliver high-quality care [71]. Job satisfaction and staff retention are key components of workforce resilience.

Although the NMWS-MMR association is most pronounced in LMICs, the issue is not confined to low-resource settings. High-income countries also face challenges. For instance, the United States has poorer maternal outcomes compared to other wealthy nations. Contributing factors include the underutilization of midwifery-led care and fragmented maternity systems [72]. International evidence suggests that work environment improvements can enhance the care quality and provider retention, thus benefiting maternal outcomes across all income settings [48],[73].

## 5. Study strengths and limitations

This study presents several methodological strengths in examining the impact of the NMWS on the MMR. The use of a delayed impact analysis enabled an exploration of long-term workforce effects, while the ecological study design allowed for the inclusion of multiple confounding variables, such as economic affluence, fertility, and urbanization, thus offering a broader understanding of the MMR determinants that is often difficult to achieve in individual-level research.

Nevertheless, key limitations should be considered. The data were sourced from global organizations such as the World Bank and the WHO. While these are reputable sources, they may contain imprecision and inconsistencies that affect the reliability of the estimates. The reliance on correlation-based methods limits the ability to draw causal conclusions. Additionally, the study risks ecological fallacy, as the population-level results may not reflect the individual-level relationships.

Furthermore, the lack of disaggregated data prevented an analysis of specific subgroups within the nursing and midwifery workforce. Information on those working exclusively in maternal health or regional variations in training and competency was unavailable. These differences may influence outcomes but cannot be fully evaluated. Another constraint is the aggregation of nurses and midwives into a single category, which makes it difficult to assess their distinct contributions despite differing scopes of practice. Future datasets should separate these professional groups to enable more a precise analysis of their individual impacts on maternal outcomes.

Overall, while the findings are robust, they should be interpreted with caution and used to guide further research that can address these gaps.

## 6. Conclusions

This study provided strong global evidence that expanding the NMWS is a critical strategy to reduce maternal mortality. A higher NMWS was consistently linked to lower MMR, even after adjusting for economic affluence, the TFR, and urbanization. The association was particularly strong in LMICs, where workforce shortages are the most severe.

Investing in the NMWS improves maternal outcomes and strengthens the overall health system capacity. However, increasing the workforce numbers alone is not sufficient. Structural supports such as professional autonomy, quality education, fair regulation, and positive working environments are essential to ensure high-quality care and staff retention.

Additionally, this study highlighted the significant influence of economic affluence. Even after adjusting for the workforce size and demographic factors, the GDP per capita remained a strong determinant of the maternal outcomes. Addressing structural and resource-based disparities is essential to achieve equitable progress in maternal health.

Strengthening the nursing and midwifery workforce through both expansion and enabling policy reform is vital. This approach not only improves maternal health but also contributes to more equitable, resilient, and effective healthcare systems globally.

## 7. Implications for nursing and midwifery authority

This global study confirms the importance of the NMWS as a policy lever to reduce maternal mortality. While workforce expansion is essential, particularly in low- and middle-income countries, supportive environments which enable autonomy, regulation, and scope of practice are equally crucial. Downe's international research highlights how systemic barriers, such as restrictive policies and fragmented care, hinder a midwives' ability to provide safe, respectful care. Her findings support midwifery-led continuity models that improve outcomes by fostering trust, dignity, and relationship-based care across the maternity continuum [72].

Structural barriers such as underfunded education systems, restrictive licensing, and negative practice environments contribute more to job dissatisfaction and attrition than workload alone [48],[73],[74]. To achieve meaningful and sustained improvements in maternal health, comprehensive reforms must priorities supportive policies, targeted education funding, and regulatory flexibility. These changes will empower nursing and midwifery professionals to lead improvements in care quality, retention, and maternal health outcomes globally.

## Use of AI tools declaration

The authors declare they have not used Artificial Intelligence (AI) tools in the creation of this article.
